# Preclinical Study of Biphasic Asymmetric Pulsed Field Ablation

**DOI:** 10.3389/fcvm.2022.859480

**Published:** 2022-03-24

**Authors:** Shengyu Bi, Fenglin Jia, Chang Lv, Qiang He, Xinyu Xu, Zhixiao Xue, Siying Su

**Affiliations:** ^1^School of Biomedical Engineering and Technology, Tianjin Medical University, Tianjin, China; ^2^Department of Cardiology, Tianjin First Central Hospital, Tianjin, China; ^3^Tianjin Intelligent Health Medical Co., Ltd., Tianjin, China

**Keywords:** pulsed field ablation, pulmonary venous isolation, atrial fibrillation, tissue-selective ablation, biphasic asymmetric pulse

## Abstract

Pulsed field ablation (PFA) is a novel method of pulmonary venous isolation in atrial fibrillation ablation and is featured by tissue-selective ablation. Isolation is achieved via the application of high-voltage microsecond pulses that create irreversible perforations in cell membranes (i.e., electroporation). We proposed a new biphasic asymmetric pulse mode and verified the lesion persistence and safety of this mode for pulmonary vein ostia ablation in preclinical studies. We found that biphasic asymmetric pulses can effectively reduce muscle contractions and drop ablation threshold. In the electroanatomic mapping, the ablation site showed a continuous low potential area, and the atrium was not captured after 30 days of pacing. Pathological staining showed that cardiomyocytes in the ablation area were replaced by fibroblasts and there was no damage outside the ablation zone. Our results show that pulmonary venous isolation using the biphasic asymmetric discharge mode is safe, durable, effective, and causes no damage to other tissues.

## Introduction

Pulsed field ablation (PFA), also known as irreversible electroporation, has been applied to cardiac ablation in recent years (1–3). PFA is a non-thermal ablative modality. It is used to treat atrial fibrillation and does not cause damage to other tissues such as the esophagus and nerves, because the threshold for cardiomyocytes is the lowest of any other tissue (4–9).

Due to these potential advantages of PFA, the technique has attracted more attention in the field of ablation treatment of atrial fibrillation (10). Although the safety and effectiveness of PFA have been proved in many experimental studies (11, 12), PFA is still in the preliminary research stage and the optimal pulse modes and ablation dose remain unclear.

The ablation effect of PFA is greatly influenced by the pulse amplitude (*y*-axis) and pulse width (*x*-axis). Lavee et al. (13) were the first to perform cardiac PFA with a monophasic direct current pulse sequence of 1,500–2,000 V, 100 μs (microsecond) per pulse, and frequency of five pulses/second. However, an electrical pulse creates local and systemic muscle contractions that make it difficult to accurately perform ablation; to solve this problem, substantial doses of chemical paralytics need to be administered to patients. Arena et al. (14) proposed a new type of high-frequency biphasic pulse to reduce muscle contraction during ablation, but the high frequency is often accompanied by heat generation. Moreover, biphasic pulses require higher voltage to achieve a similar effect compared with monophasic pulses, because the cancellation effect means that the effect of the first pulse is reduced by the second pulse of opposite polarity (15, 16). For example, Sano et al. (17) found that the lethal threshold of a biphasic symmetric pulse was 1,316 V/cm, which was significantly higher than that of a biphasic asymmetric pulse (536 V/cm). Our previous study on smooth muscle cells and cardiomyocytes found that asymmetric pulse width was superior to biphasic symmetric pulse ablation under equal amplitudes (18).

Based on our previous cell experiment, PFA with biphasic asymmetric pulses was carried out in 12 Bama miniature pigs. Gross examination and histological investigation were used to evaluate the lesion persistence and safety of PFA at the 7th and 30th day after pig PFA operation. Moreover, PFA with biphasic asymmetric pulses was carried out in 2 dogs, and electroanatomic mapping was used to display the lesion area. Our results would be used to help to make a treatment plan for clinical trials in the future.

## Materials and Methods

### Materials

#### Experimental Animals

Twelve healthy 12-month-old Bama miniature pigs (weight 45 ± 5 kg; of either sex) were provided by Tianjin TEDA Cardiovascular Hospital-Animal Experiment Center (SYXK (JinBin) 2020-0004). Two healthy Labrador retrievers (weight 24 and 25.1 kg; of either sex) were provided by Beijing Tonghe Litai Biotechnology Co., Ltd. (SCXK (Jing) 2019-0005). All our experiments were approved by the Ethics Committee (approval number:2021021).

#### Reagents and Instruments

The medicine used in the present study were Sumianxin II injection (compound preparation of Jingsongling, edetic acid, Dihydroetorphine hydrochloride (DHE), and haloperidol) (Jilin Dunhua Shengda Animal Pharmaceutical Co., Ltd.), 1% propofol injection (AstraZeneca, Italy), and enoxaparin sodium injection (Sanofi, France). Other medicines and instruments were conventional equipment found in laboratories and catheterization laboratories.

### Experimental Method

#### Preoperative Treatment

The animals were fasted for 12 h before operation, and water was withheld for 6 h. Aspirin (5 mg/kg) was administered once daily for the first 3 days. Before operation, blood was collected from the precaval vein for preoperative routine blood testing. Conventional doses of xylazine and midazolam were used for the induction of anesthesia. After the peripheral venous access was established, 30–50 mg of propofol was intravenously infused as appropriate to obtain stable anesthesia. After being weighed, the animals were placed in a *U*-shaped groove in supine position on the digital subtraction angiography operating bed, and tracheal intubation was performed. A ventilator was used to support mechanical ventilation. The skin on the front of the chest and the bilateral inguinal regions was prepared for operation. The bilateral femoral veins were punctured by Seldinger's method and a 6F vascular sheath was placed. Propofol was infused at a rate of 3–5 mg/kg/h throughout the operation to maintain anesthesia. The vital signs were routinely monitored intraoperatively.

#### Pulsed Field Catheter Ablation

After the atrial septal puncture sheath was guided into the left atrium under endoluminal sonography and X-ray fluoroscopy, 6,000 units of heparin were injected, and 1,000 units of heparin were added every hour during the operation. The position of the sheath was adjusted, and pulmonary venography was performed to show the shape and branching of the pulmonary veins. A circular mapping catheter was placed in the left superior pulmonary vein to evaluate the effect of electrical isolation.

**Figure 1:**
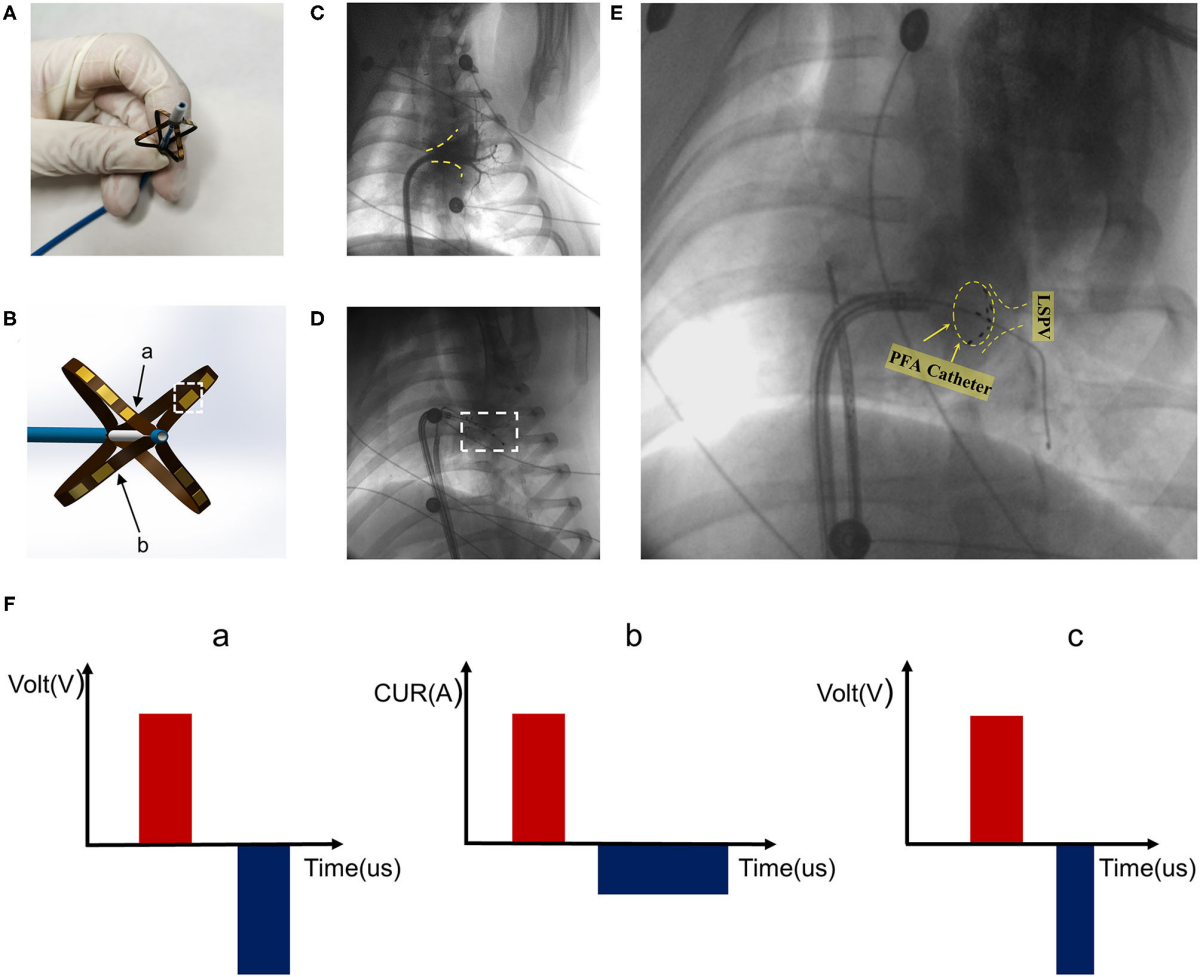
Pulsed field ablation system. **(A)** Biphasic asymmetric pulsed field ablation catheter in open state. **(B)** Illustration of the front end of the ablation catheter. Arrow a is the positioning electrode. Arrow b refers to the catheter frame. The white dotted box represents the electrode. **(C)** Left superior pulmonary angiography. Yellow dotted line marking the contour. **(D)** Pacing after ablation. **(E)** PFA catheter for ablation at the left superior pulmonary vein ostia. PFA catheter shape shown in yellow dotted circle. **(F)** Pulsed field ablation mode. Mode c is biphasic asymmetric pulsed field ablation.

The PFA system was composed of a pulse instrument (PFA instrument) (Tianjin Intelligent Health Co., Ltd., Tianjin, CHN) and a PFA catheter (Tianjin Intelligent Health Co., Ltd., Tianjin, CHN) (Figure 1A). The pulse instrument was used to set the parameters, including the number of pulses, number of pulse groups, and pulse amplitude. The 10.5 F PFA catheter had four frames; each frame contained two electrodes, one of which was a positioning electrode (Figure 1B). When fully expanded, the diameter of the most distal electrode was 28 mm.

The PFA catheter was half-opened in the left atrium through a flexible sheath, and then with the guidance of guidewires, the ablation electrode was pushed to a position close to the pulmonary vein ostium. The adherence condition was reflected by angiography and signal feedback of the PFA instrument (Figure 1E). The catheter electrode was optimized, and the pulse mode was modified. As shown in Figure 1F, based on the existing two pulse modes a and b, the pulse mode was improved to type c (Figure 1F, c), so that the negative narrow pulse depolarized the positive wide pulse and reduced the muscle contractions caused by the pulses, which enhanced the safety and maneuverability. The pulse width of all positive pulses was 5 μs and the pulse width of negative pulses was 3 μs. The pulse voltage, pulse width, frequency, and electrode spacing of the PFA system used in the present study were able to be adjusted to ensure the ablation effect. The pulse released 1,000 V microsecond pulses through the ECG synchronization signals.

The experiment included two groups. Group A and B comprised six pigs each. PFA was carried out in all pigs. Animals in Group A were euthanized at 7th day after ablation and in Groups B at 30th day after ablation. The ablation tissue (left superior pulmonary vein) was regarded as the experimental group and non-ablation tissue (left inferior pulmonary vein) was the control group. After electric discharge, pacing was performed by a pacemaker (Medtronic 5388, MN, USA) in groups A and B. The pacing voltage was 10 mv, and the pacing frequency was about 15% higher than the heart rate before ablation (Figure 1D). As the biphasic asymmetric pulse method greatly reduced the impact on the skeletal muscles, no intervention was performed for the skeletal muscles.

Besides, in order to further verify the effectiveness of PFA, two dogs were selected as dog group and electroanatomic voltage mapping of the left atrium was constructed after ablation.

#### After Care

All animals underwent Computed Tomography (CT) to check for adverse conditions such as air emboli, thrombi, vascular access tears, and cardiac tamponade. After the catheter was withdrawn, the condition of the puncture points was observed. The vital signs, mental behavior, and activity status of the animals were observed after they awoke from anesthesia.

Animals were injected with 20 IU/kg of intramuscular penicillin sodium + 0.9% normal saline twice daily for 3 days after operation to prevent infection. Intensive care was performed after operation, and the clinical changes were recorded. Daily wound cleaning was implemented to prevent infection. On post-op days 7 and 30, twelve pigs were euthanized and dissected for gross observation and pathological analysis. The dogs were followed up for 30 days, and circular mapping electrodes were used to create electroanatomic maps on each animal.

#### Gross Examination of Specimens

The surviving pigs were euthanized on post-op days 7 (*n* = 6) and 30 (*n* = 6). The heart, lungs, and adjacent trachea and esophagus were removed as a whole. The hearts were dissected, the ablation sites were identified, and the pathological changes of the ablation sites and adjacent tissues were observed. The continuity of the ablation area and the presence of local thrombosis were evaluated.

#### Histological Investigation

The specimens were fixed in formalin and stained with Masson trichrome and HE to evaluate the presence of lesions in the pulmonary vein ostia, transmural ability, neurological activity in the lesions, presence of thrombi and edema, degree of damage to adjacent tissues, and other related findings. Other assessed variables included fibrosis, inflammation, hemorrhage, and nerve damage.

#### Statistical Analyses

Origin8.5 software was used for data analysis. The data were presented by Average ± SD or Median (25th, 75th percentiles). The Mann-Whitney U test was used for continuous parameters in different experimental and/or control groups due to the non-normal distribution and the small sample size. *p* < 0.05 was defined as statistically significant.

## Results

### Clinical Observation and Survival Rate

The first day after operation, the behavioral activities, mental status, appetite, and food intake of the animals returned to normal. The animals had no hollow back, piloerection, loose stools, or perinasal bleeding throughout the experiment. No animals died, and the blood test results were normal on the 7th and 30th day after ablation.

### Acute Experiments

The ablation zone was the left superior pulmonary vein in all pigs, with an ablation success rate of 100% (12/12). In the pigs (Group A&B), the average ablation times were 84.06 ± 13.09 s, and the average operation time was 92.3 min. The average peak current of ablation in the pig experiment was 7.83 ± 1.90 A (Ampere). The ablation zone was the right superior pulmonary vein in all dogs, with an ablation success rate of 100% (2/2). In the dog group, the average ablation time for 1,000 pulses was 71.90 ± 2.48 s. The average peak current of ablation in the dog experiment was 7.10 ± 0.42 A (Table 1). None of the animals showed obvious arrhythmia during the experiments.

**Table 1:** Procedural characteristics of biphasic asymmetric PFA.

**Group**	**A&B**	**Dog group**
PFA Times, median (sec)	81.6 (73.7, 94.3)	71.9 (71.0, 72.8)
Peak current, average (Amp) ±SD	7.83 ± 1.90	7.10 ± 0.42
Therapy dose (V, Voltage)	1,000 V	1,000 V
Procedure time, median (minutes)	95 (88, 104.5)	81.5 (80.75, 82.25)

*PFA Times data are presented as median (25th, 75th percentiles)*.

*Procedure time data are presented as median (25th, 75th percentiles)*.

*PFA, pulsed field ablation; sec, second; Amp, ampere; SD, Standard Deviation*.

**Figure 2:**
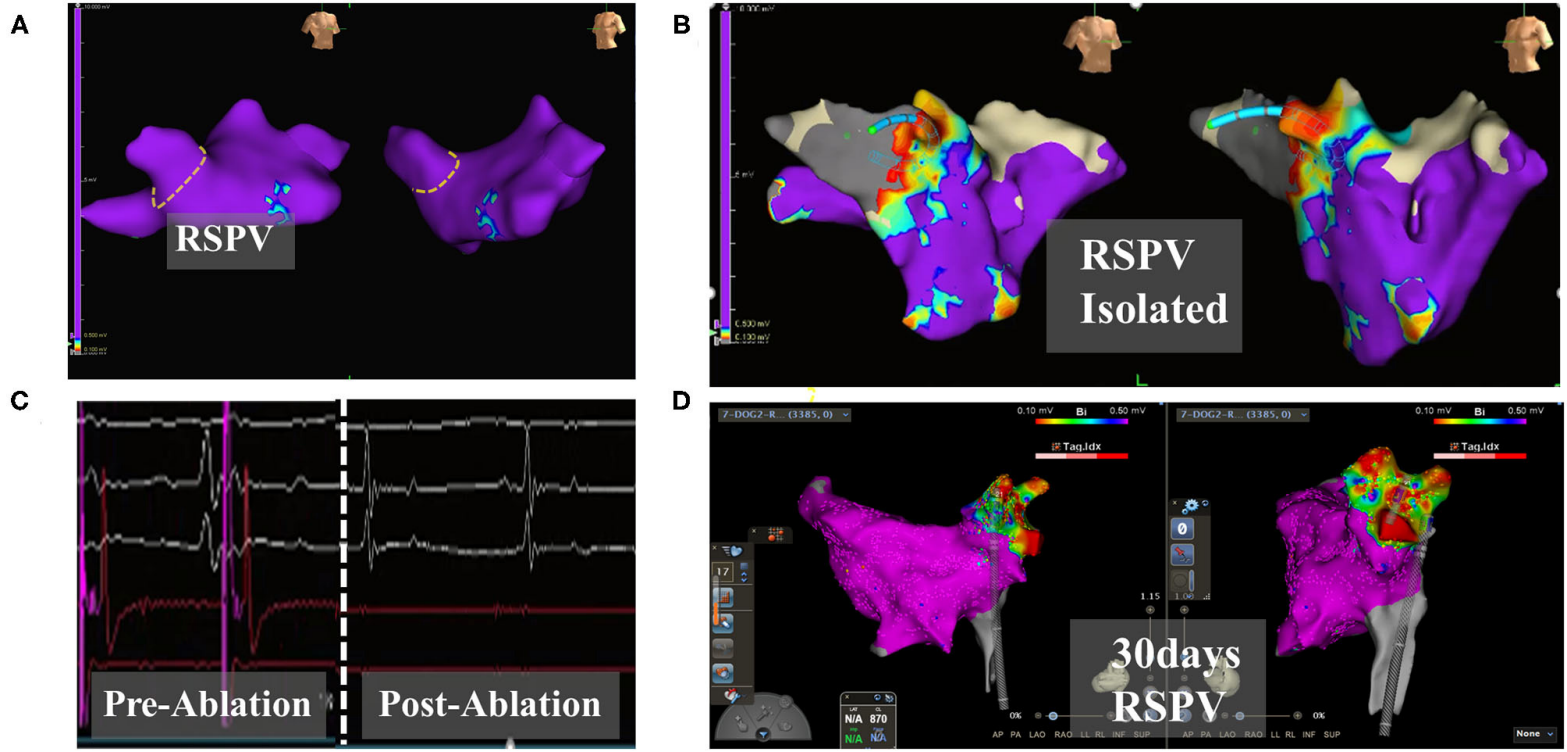
Electroanatomic modeling of the right superior pulmonary vein. **(A)** Baseline potentials in right superior pulmonary were assessed. The yellow dotted line draws the right superior pulmonary vein ostia. **(B)** Post-operative ablation site shows a low potential area. **(C)** Pulmonary vein electrograms from PFA catheter ablation. Left: Pre-ablation. Right: Post-ablation. **(D)** Right superior pulmonary vein potential after 30 days. Continuous low potential in the ablation zone. The color of the mapping from red to purple indicates the potential from low to high. EnSite, Abbott: **(A–C)**. Carto3, Johnson & Johnson: **(D)**.

When the pigs were given pacing stimulation after the ablation, the pacing signal was detected by ECG monitoring, but the heart rate did not increase. In the dog group, electroanatomic mapping showed that the right superior pulmonary vein potential disappeared immediately after ablation (Figures 2A,B). The voltage mapping showed low voltage, indicating afferent block (Figure 2C). At the 30th day after operation, the right superior pulmonary vein pacing did not cause atrial capture, indicating afferent block (Figure 2D).

### Gross Observation of the Pulmonary Vein Ostia and Adjacent Tissues

After all pigs were euthanized, gross observation was conducted to identify any esophageal or tracheal deformation and to evaluate the smoothness of the surface. The conditions of the adventitia and epithelium of the esophagus and the conditions of the tunica adventitia of trachea were observed with the naked eye and found there was no damage. The lung specimens were soft, light, smooth, moist, elastic, spongy, and free of scarring. The vagus nerves were intact and the upper and lower connections were normal under observation with the naked eye (Figure 1).

**Figure 3:**
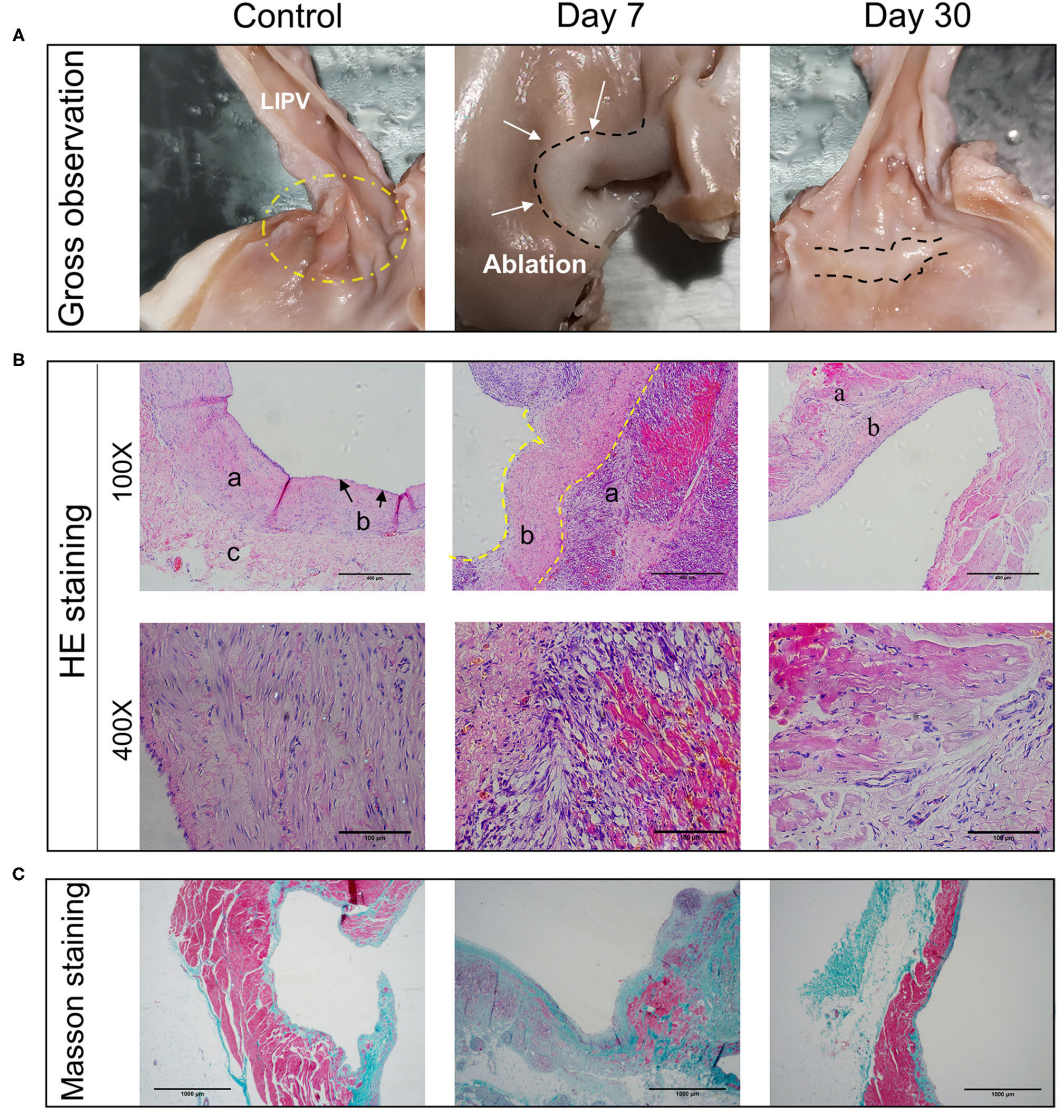
Pathological changes of myocardial tissues after PFA ablation. **(A)** Gross observation of the left superior pulmonary vein ablation site. In the control group, lesion areas were not seen. The 7- and 30-day groups (group A and B) show a clear ablation zone. **(B)** HE staining of ablation sites at 100X and 400X. a indicates myocardial tissue. b represents intimal layer (endothelium, fibroblasts and connective tissue). c is marked with adventitia. **(C)** Masson staining of ablation sites (40X). Control group, days 7 and 30 from left to right.

On the 7th day after operation, the ablation zones had a pale circular pulmonary vein appearance with a clear boundary (Figure 3A, Day 7). On the 30th day after the operation, there was an obvious white circular ablation zone in the pulmonary vein ostia, with similar morphology as on the 7th day after operation (Figure 3A, Day 30). There was no obvious damage outside the ablation zone. The boundary was clear, the transmural ability was obvious, and there was no pulmonary vein stenosis or thrombosis (Table 2).

**Table 2:** Pathology summary of Group A and B.

**Group**	**A**	**B**	*P*-value^*^
Number of ablated locations (*n*, total)	6	6	–
Targeted anatomical location	LSPV	LSPV	–
Euthanasia (d, days)	7 d	30 d	–
Transmurally achieved	6/6	6/6	–
Wall thickness of ablated tissue, average (mm) ± SD	0.68 ± 0.24	0.60 ± 0.27	0.589
Thickness of Cf, average (mm) ± SD	0.68 ± 0.24	0.096 ± 0.03	0.002
Depth of Cf (median, %)	100.00%	18.30%	0.002
Other lesions	0/6	0/6	–

* *P-values were shown for the Mann-Whitney U-test*.

*LSPV, left superior pulmonary vein; RSPV, right superior pulmonary vein; Cf, collagen fibers*.

*The total wall thickness in the control group was 0.88 ± 0.02 (average ± SD)*.

*The collagen fiber thickness in the control group was 0.11 ± 0.04 (average ± SD)*.

### Histological Investigation

At 7 days after the operation, there was a clear circumferential ablation zone at the ablation site. Under 100× magnification, HE (hematoxylin-eosin) staining showed a clear boundary between the ablation and non-ablation zones and a large amount of layer-by-layer deposition of collagen and fibrotic tissue proliferation under the intima of the ablation zone; the myocardial cells had irregular morphology and were arranged in a disorderly fashion (Figure 3B, 100X, Day 7). Under 200× magnification, there was increased infiltration of monocytes, a certain amount of cell infiltration and proliferation of epithelioid cells, and hyperemia in the middle layer of the myocardial tissue. Under 400× magnification, the nuclei of infiltrating cells were as oblong as those of smooth muscle cells (Figure 3B, 400X, Day 7).

At 30 days after the operation, the infiltration of the middle cardiac muscle layer had disappeared, and there was no congestion, inflammatory cells, or signs of thrombosis. The middle layer of cardiomyocytes had shrunk in shape, the nuclei had fragmented or disappeared, cardiomyocytes showed a large amount of necrosis, myocardial endothelial cells had formed, and a large number of fibroblasts had replaced cardiomyocytes and were growing under the endothelium. There were no obvious effects on arterioles and venules (Figure 3B).

Masson staining of specimens collected on the 7th post-operation (post-op) showed a thickened intimal layer of the pulmonary vein, a large number of collagen fibers. The average thickness of collagen fibers is 0.68 ± 0.21 (±SD). And a large amount of collagen in the intimal medial layer (Figure 3C, Day 7). At 30 days, the number of collagen fibers in the intimal layer had basically returned to normal (Figure 3C, Day 30), 0.096 ± 0.03 (±SD) (Table 2). Using the Mann-Whitney *U*-test, there is significant differences in the variable of transmural ability indicated by percentages of the collagen fiber depth at the 7th day after operation (*P* = 0.002), compared with the control (Table 3). However, the measurement of transmural ability appeared similar to the control on the 30th day after operation (*P* = 0.699).

**Table 3:** Comparisons for percentages of the collagen fiber depth over total thickness.

**Groups**	Depth percentage^*^	***P*-value**
**7 d after the operation**		
Group A (*N* = 6)	100% (100%, 100%)	0.002
Control A (*N* = 6)	13.27% (11.28%, 14.95%)	
**30 d after the operation**		
Group B (*N* = 6)	18.30% (13.68%, 20.14%)	0.699
Control B (*N* = 6)	16.01% (11.30%, 19.04%)	

* *Depth Percentage means the percentages of the collagen fiber depth over total thickness, and they are presented as median (the 25th percentile, the 75th percentile)*.

## Discussion

The usual atrial fibrillation catheter ablation is performed using the RF technique, which applies a high-frequency alternating current to heat and damage the myocardial tissues (19). However, the disadvantages of the RF technique are the difficulty in controlling the size of the treatment zone and the high risk of adverse effects due to excessive damage. Subsequently, the cryoballoon ablation technique emerges. The cryoballoon technique is the use of cryogenic energy, which leads cells to necrosis by freezing. However, it damages blood vessels and other tissues (20). PFA technology is derived from the principle of electroporation, which exposes cells to electrical pulses to increase the permeability of cell membranes (21). Previous studies have shown that cardiomyocytes have lower electroporation thresholds than cell types related to collateral damage, such as the cells of nerves, arteries, and the esophagus. For example, Kaminska et al. (22) found that a pulse intensity higher than 375 V/cm had an ablation effect on cardiac muscle in a MTT assay on rat cardiomyocytes. Koruth et al. (23) found no lesions in the lumen and outer surface of the esophagus after ablation with a peak electric field intensity of 900 V/cm during PFA of the esophagus. Maor et al. (7) found that the ablation of smooth muscle cells was ineffective when the electric field intensity of the pulse field was lower than 875 V/cm. Due to differences between cell types in electroporation thresholds, the unique tissue selectivity of PFA in the treatment of atrial fibrillation has the great advantage of reducing complications compared with ablation via traditional energy sources, such as RF, which indiscriminately damage all tissues in the hot zone. In clinical operation, isolation using the RF method is performed in a point-by-point mode. The mode needs further mapping and ablation. In contrast, PFA creates a closed-loop isolation region, which avoids the problem of RF ablation and greatly shortens the ablation time.

Preclinical studies have evaluated parameters that affect the effectiveness and safety of PFA, such as the electric field intensity and pulse field direction (24). And Reddy et al. had already demonstrated that PFA preferentially affected myocardial tissue with excellent durability and chronic safety in human trials (11). The mode of ablation in (11) was biphasic and symmetric. The difference between our experiment and previous PFA studies is the introduction of a new biphasic asymmetric pulse ablation mode, which narrows the width of the negative pulse and reduces the contractions of the muscle caused by the positive pulse (18). So before the clinical experiments, pigs were used to evaluate the safety and efficacy of biphasic asymmetric pulse ablation mode. As a result, there is no need to inject muscle relaxant before operation. In the original biphasic symmetric pulse mode (Figure 1F, a) (14), there was a certain amount of cancellation, which required the provision of a higher pulse intensity during ablation (25). The biphasic asymmetric pulse can reduce muscle contraction compared to monophasic pulses, and has the advantage of lower cell ablation threshold compared to biphasic symmetric pulses. We also updated the catheter used in previous experiments (18), and added positioning electrodes to the new catheter to ensure that the current state of electrodes was displayed under X-ray to provide more reliable information to doctors. Furthermore, we reinforced the framework of electrodes to make them more rigid and increase the stability of the electrode structure.

In the present study, the ablation results were recorded at 1 and 4 weeks, and the lasting effectiveness and safety of the biphasic asymmetric PFA system in the treatment of atrial fibrillation were verified, ensuring the safety of future clinical trials using the same system. In the pig experiment (Group A&B), there was no ventricular arrhythmia at any time during the operation, and the behavioral activities, mental status, appetite, and feed intake of the pigs returned to normal after the operation. We examined the near-ablation zone, including the vagus nerve, lung, trachea, and esophagus. A pathological analysis of the ablation zone performed at 1 and 4 weeks showed that all targeted parts produced complete transmural lesions and maintained good safety. At 4 weeks after PFA, fibroblasts had replaced the original cardiomyocytes and PFA had not destroyed the intercellular connection, which was conducive to the maintenance of the tissue structure. There was no thromboembolism, intracardiac injury, or acute or chronic collateral tissue injury after ablation. Consistent with our previous *in vitro* experiments (18), the present results further proved that the biphasic asymmetric pulse mode and PFA system used in the present study had good safety and produced effective lesions. In dog group, the immediately post-op ablation zones showed a low potential and a ring shape. The electrical signal level measured at 4 weeks after ablation showed that the potential remained low. Pacing did not capture atrium, and it confirmed the effectiveness of ablation and the safety of the experimental animals. More details in Supplementary Figure 2.

The number of pulses in the pulse sequence is an important parameter, and there is currently no clear consensus on the optimal number of pulses (26). Stewart et al. (27) set the number of pulses to a group of 60 (six pulses released per heartbeat), while Koruth et al. (23) proposed a pulse sequence with five pulses per group. We found that when the number of pulses was set to 20 (all pulses released after one R wave), from the 15th pulse, a current amplitude peak appeared which was much higher than the normal amplitude value. This phenomenon may be caused by an unstable heart rate. Alternatively, when the number of pulses is sufficient, a large amount of intracellular fluid may overflow from the tissue cells, increasing the conductivity and sharply increasing the current amplitude. When we reduced the number of pulses, the current returned to its normal value. We believe that this was due to the self-recovery function of tissue cells in a short period of time. The existence of this phenomenon allowed us to keep the number of pulses in each group to 10 (similar to the parameters used in previous studies). The length of the blank period between the positive and negative pulses is generally referred to as the pulse interval. There is no standard formula for determining the length of the pulse interval (16, 28). In subsequent studies, we plan to study the effect of the pulse width and the pulse interval on the ablation.

PFA is one kind of AF catheter ablation, so the drug treatment after the PFA can be referenced to the guideline of 2020 ESC Guidelines for the diagnosis and management of atrial fibrillation developed in collaboration with the European Association for Cardio-Thoracic Surgery (EACTS) (29). In the future clinical trials, we will investigate the interaction between the drug and PFA.

## Limitations

In this experiment, the novel PFA catheter had high levels of safety and durability. However, the only parameter setting that was varied in this experiment was the number of pulses, while the other parameters of the PFA system were kept constant. The complex intracardiac environment and local dynamic electrical characteristics of tissues will have a certain impact on the PFA zones. Considering the difference of heart between human and animal, the distance between electrodes should be adjustable. So the voltage amplitude and the number of pulses should be adjusted during the ablation process in order to achieve a high degree of durable pulmonary vein isolation.

## Data Availability Statement

The original contributions presented in the study are included in the article/Supplementary Material, further inquiries can be directed to the corresponding author/s.

## Ethics Statement

The animal study was reviewed and approved by Tianjin TEDA Cardiovascular Hospital-Animal Experiment Center.

## Author Contributions

ZX and XX: conceptualization, writing—review and editing, and funding acquisition. SB, SS, and QH: methodology and investigation. QH: clinical operation. SB and FJ: data curation. SB and CL: writing—original draft preparation and visualization. All authors have read and agreed to the published version of the manuscript.

## Funding

This research was funded by Tianjin Science and Technology Committee (Grant Nos. 19ZXYXSY00050 and SQ2020YFF0406649) and Tianjin Municipal Health Bureau (Grant No. ZC20165).

## Conflict of Interest

SS was employed by Tianjin Intelligent Health Medical Co., Ltd. The remaining authors declare that the research was conducted in the absence of any commercial or financial relationships that could be construed as a potential conflict of interest.

## Publisher's Note

All claims expressed in this article are solely those of the authors and do not necessarily represent those of their affiliated organizations, or those of the publisher, the editors and the reviewers. Any product that may be evaluated in this article, or claim that may be made by its manufacturer, is not guaranteed or endorsed by the publisher.
