# Supplementary material for: Preclinical Study of Biphasic Asymmetric Pulsed Field Ablation
Source: Front Cardiovasc Med. 2022 Mar 24;9:859480. doi: 10.3389/fcvm.2022.859480 (PMC8987372; doi:10.3389/fcvm.2022.859480)
Supplement: Supplementary file 4 [file Data_Sheet_1.pdf]

Supplementary Material

1 Supplementary material for Group A&B

1.1 Gross observation and HE staining of the adjacent tissues

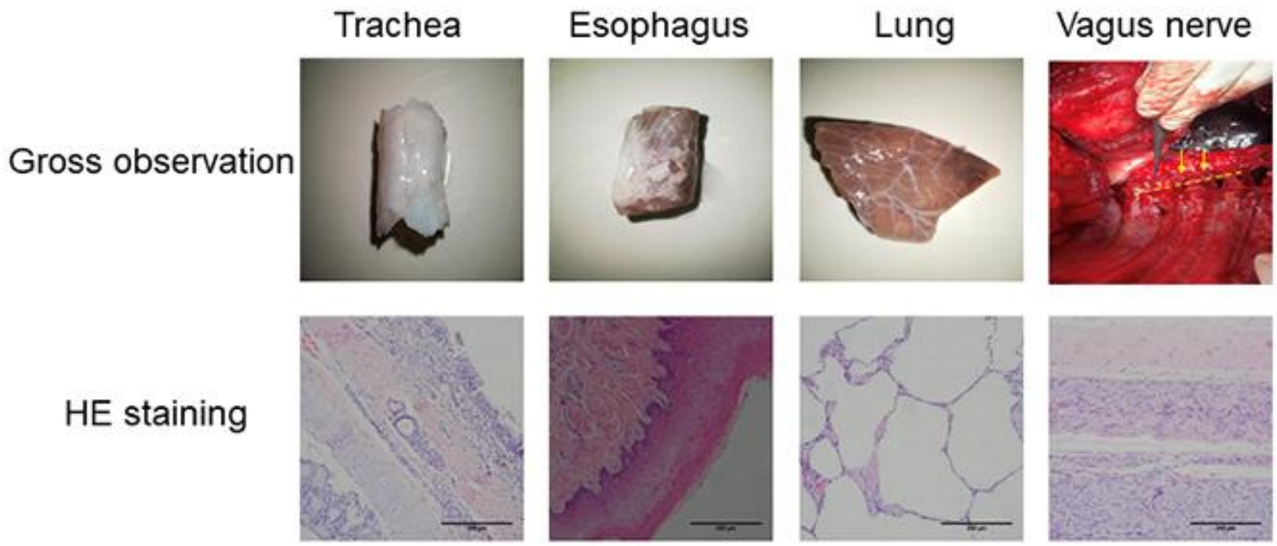

Supplementary Figure S1. Other tissues adjacent to PFA ablation site after 7 days. Trachea adjacent to the ablation site, we did not see the damaged area caused by the ablation of the pulse field. The adventitia, hyaline cartilage, submucosa and lamina propria were not damaged. No scar on the outside of the esophagus. Staining of esophageal cross section from outside to inside. The cells in the muscular and mucosal layers were normal, and no esophageal fistula was observed. A cut of lung tissue was soft, light and smooth, pathology of the lungs near the left atrium. Near ablation end of the vagus nerve segment (The black dotted line and arrow indicate the vagus nerve). No lesions on the left vagus nerve. All pictures were observed under a 200x microscope**.**

1.2 Supplementary Table S1: Blood test report

Due to the large amount of content, it has been placed in Table S1 bloom testing. We take blood test before, after, 7 days after and 30 days after the operation and have the blood checked. And we need to focus on is the Attention page. These include CK, CK-MB and CREA etc. This is important for PFA.

1.3 Supplementary Video S1: Pulsed field ablation process

This video shows the pulsed field ablation process, including the start of the procedure, fluoroscopy and pulse releases.

1.4 Supplementary Video S2: Digital subtraction angiography for pulsed field ablation

The video consists of three parts. The first part is pulmonary venography, the second part is pulsed field ablation catheter placed in target tissue for ablation, and the third part is electrical stimulation to verify whether the ablation is successful or not.

2 Supplementary material for dog experiment

2.1 Pulsed field ablation process

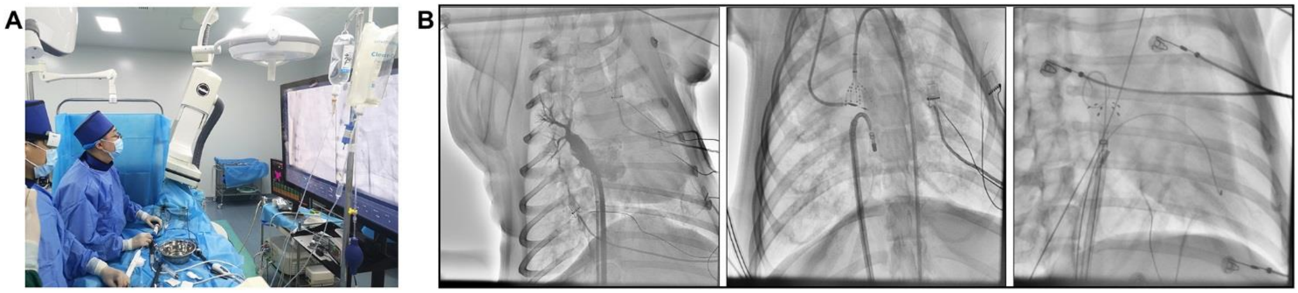

Supplementary Figure S2. A The Dog's Operation in Progress. **B** X-ray images, including right superior pulmonary angiography, PFA delivery and Carto system mapping.

2.2 HE staining and Masson staining of target tissues

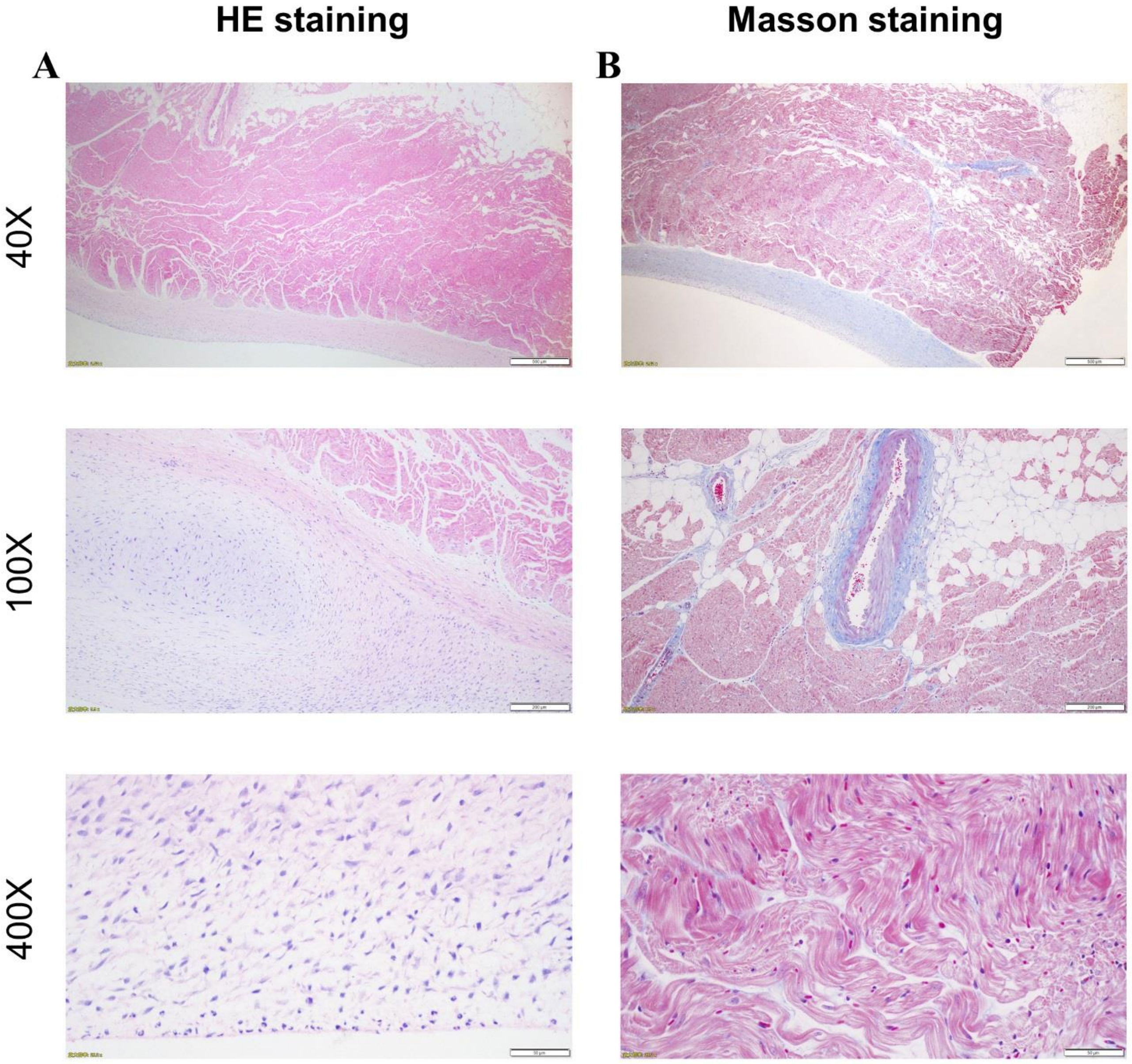

Supplementary Figure S3. A HE staining. **B** Masson staining. Staining showed that the endothelium was missing, and the depth reached the intimal layer. Eosinophils gathered locally in the intimal layer (The images were observed under a 40x, 100x and 400X microscope from up to down).
